# Early Transcriptomic Response to LDL and oxLDL in Human Vascular Smooth Muscle Cells

**DOI:** 10.1371/journal.pone.0163924

**Published:** 2016-10-11

**Authors:** Salvador Damián-Zamacona, Paola Toledo-Ibelles, Mabel Z. Ibarra-Abundis, Laura Uribe-Figueroa, Enrique Hernández-Lemus, Karla Paola Macedo-Alcibia, Blanca Delgado–Coello, Jaime Mas-Oliva, Juan Pablo Reyes-Grajeda

**Affiliations:** 1 Instituto de Fisiología Celular, Universidad Nacional Autónoma de México, México City, México; 2 Instituto Nacional de Medicina Genómica, México City, México; University of Sassari, ITALY

## Abstract

**Background:**

Although nowadays it is well known that the human transcriptome can importantly vary according to external or environmental condition, the reflection of this concept when studying oxidative stress and its direct relationship with gene expression profiling during the process of atherogenesis has not been thoroughly achieved.

**Objective:**

The ability to analyze genome-wide gene expression through transcriptomics has shown that the genome responds dynamically to diverse stimuli. Here, we describe the transcriptome of human vascular smooth muscle cells (hVSMC) stimulated by native and oxidized low-density lipoprotein (nLDL and oxLDL respectively), with the aim of assessing the early molecular changes that induce a response in this cell type resulting in a transcriptomic transformation. This expression has been demonstrated in atherosclerotic plaques *in vivo* and *in vitro*, particularly in the light of the oxidative modification hypothesis of atherosclerosis.

**Approach and Results:**

Total RNA was isolated with TRIzol reagent (Life Technologies) and quality estimated using an Agilent 2100 bioanalyzer. The transcriptome of hVSMC under different experimental conditions (1,5 and 24 hours for nLDL and oxLDL) was obtained using the GeneChip Human Gene 1.0 ST (Affymetrix) designed to measure gene expression of 28,869 well-annotated genes. A fixed fold-change cut-off corresponding to ± 2 was used to identify genes exhibiting the most significant variation and statistical significance (*P< 0*.*05*), and 8 genes validated by qPCR using Taqman probes.

**Conclusions:**

10 molecular processes were significantly affected in hVSMC: Apoptosis and cell cycle, extracellular matrix remodeling, DNA repair, cholesterol efflux, cGMP biosynthesis, endocytic mechanisms, calcium homeostasis, redox balance, membrane trafficking and finally, the immune response to inflammation. The evidence we present supporting the hypothesis for the involvement of oxidative modification of several processes and metabolic pathways in atherosclerosis is strengthen by the fact that gene expression patterns obtained when hVSMC are incubated for a long period of time in the presence of nLDL, correspond very much the same as when cells are incubated for a short period of time in the presence of chemically modified oxLDL. Our results indicate that under physiological conditions and directly related to specific environmental conditions, LDL particles most probably suffer chemical modifications that initially serve as an alert signal to overcome a harmful stimulus that with time might get transformed to a pathological pattern and therefore consolidate a pathological condition.

## Introduction

Atherosclerosis is a multifactorial chronic disease characterized by the accumulation of lipids and fibrous tissue in a lesion known as atheromatous plaque; which together with myocardial infarction, one of its most frequent complications, corresponds to the first cause of death globally[1]. Likewise, a similar increase in the prevalence of hypercholesterolemia, one of the main known risk factors[2], has been well documented globally. The presence of prone to rupture plaques, thrombi formation, and ischemic processes in both coronary atherosclerotic disease (CAD) and cerebrovascular disease (CVD) can be clinically identified [3]. In the later stages of the disease, it is possible to detect foam cells along with an aberrant production of proinflammatory cytokines and growth factors which promote a phenotype active in proliferation and migration of vascular smooth muscle cells (VSMC), suggesting a transdifferentiation process[4–7].

To date, it is considered that plaque formation originates within a process involving the three layers of arteries. One of the main hypotheses about the genesis of atherosclerosis points to the process of oxidative stress as a cause of the early lesion known as a fatty streak [8], where foam cells covered by endothelial cells and VSMC of heterogeneous morphology coexist[9]. Before foam cells appear, the accumulation of low-density lipoproteins (LDL) in the subendothelial space with a high degree of oxidation is a factor accelerating the progress of the disease[10, 11], as well as the modification of the characteristics of lipoproteins altering their capacity to be normally metabolized[12–15]. The intravascular transport of these lipoproteins is a dynamic process based on conformational changes of proteins and temporary interactions within the surface of lipoproteins, both sensitive to oxidative stress[16, 17]. Lipoprotein oxidation due to exposure to reactive oxygen species (ROS) [18, 19]promotes the production of foam cells and therefore phagocytosis[20, 21]. Oxidized LDL (oxLDL), their lipoperoxides and free radicals are moved towards the cytoplasm, propagating more ROS inside the cell and perpetuating a chronic effect[22]. It seems that oxLDL also aggravate the growth of lesions by inducing the expression of adhesion molecules, synthesis of chemokines by monocytes and, in the long term, apoptosis of endothelial cells[23–25]. Employing several *in vivo* models of atherosclerosis, it has been proved that an appropriate balance of circulating antioxidant chemical species limits the progress of the disease, thus supporting the role of oxidative stress in the development of CAD[26, 27]. Although the effect of oxLDL upon endothelial cells and vessel function is well-known, little is known about the phenomena taking place in vascular layers other than the endothelium. Recently, it has been shown that the adventitia, considered a cell layer showing a low level of organization and therefore neutral regarding the development of CAD, also contributes to the repair of the vessel wall by establishing communication between endothelial and smooth muscle cells[28].

Here, we assessed the transcriptomic response of hVSMC to the exposition to both native and oxidized LDL using microarrays of the full transcriptome. We found that the *in vitro* exposure to oxidized LDL modifies the expression of an important number of genes. Remarkable changes were observed in genes related to CAD, such as those regulating inflammation, cell cycle, transcription regulation and calcium homeostasis. Our results show that at short periods of time oxLDL promotes an antiatherogenic cellular response, in contrast to results obtained under a chronic exposure to these stimuli, where cells respond with alarm signals leading towards an atherogenic phenotype.

For the first time a series of transcriptomic shifts are presented in association to the metabolism of hVSMC when exposed to oxLDL particles. Changes found in molecular nodes such as phenotype transdifferentiation, lipid metabolism regulation, and extracellular matrix remodeling among others, provide new evidence regarding the importance of vascular smooth muscle cells in the process of atherogenesis.

## Results

### Transcriptomic Data

**Fig 1:**
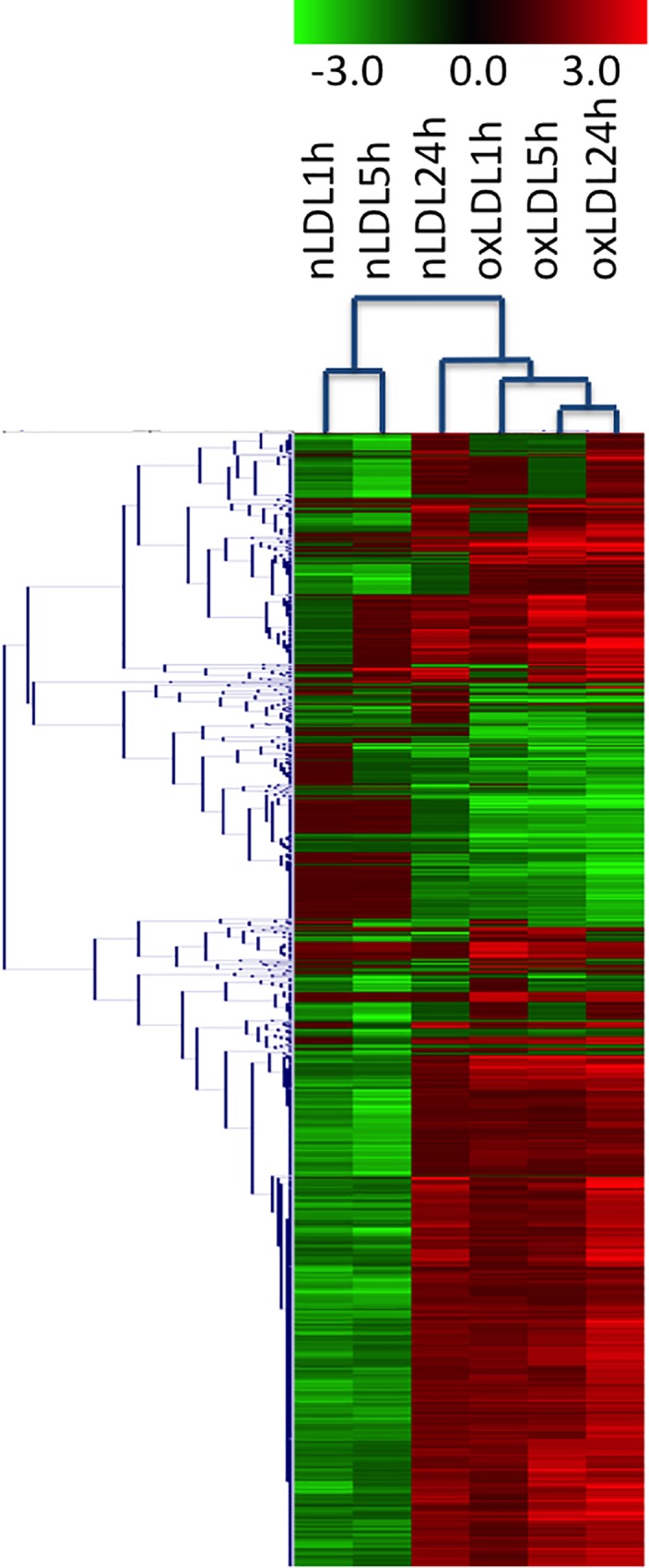
Hierarchical cluster analysis of the differentially expressed genes with more than 2-fold changed expression in one out of six groups (nLDL 1h, nLDL 5h, nLDL 24h, oxLDL 1h, oxLDL 5h and oxLDL 24h) compared to parental hVSMC cells. The dendrogram on the left indicates correlation between gene expression profiles. The columns in the middle show the normalized expression of each gene in pseudocolor scale (key in the upper).

While 236, 586 and 208 genes were differentially expressed by hVSMC exposed to nLDL at 1, 5, and 24 h respectively; in the presence of oxLDL genes showing a ± 2 fold change correspond to 231, 425 and 799 under similar incubation times. These data show that oxidative stress induced by chemically oxidized LDL and the “natural” oxidation of nLDL along time, lead to an increased activity in gene expression while the process of transcription is apparently less affected. In all conditions assessed, gene upregulation apparently is more dramatic than downregulation (Table 1). Using a bioinformatics analysis of Hierarchical clustering (Fig 1) it is evident that VSMC incubated for long periods of time with nLDL present a transcriptomic response similar to the one seen at shorter times when oxLDL are employed. Since under expressed genes are also important in the regulation of cell metabolism, we suggest that along with harm stimuli, cells initially make an effort to maintain cell homeostasis and only with time eventually make the critical decision addressed to repair damage or, for example, start an apoptotic event.

**Table 1:** Top molecular and cellular functions significantly affected by the internalization of nLDL or oxLDL carried out at different times correlated with the top canonical pathways involved.

TX	Molecular and cellular functions	Enrichment (P value)*, Molecule counts	Top canonical pathways	p-value (Molecule counts)
nLDL 1h	Cell cycle	2.81E-05–4.57E-02 (19)	CNTF signaling	8.03E-05 (5)
	DNA replication,recombination and repair	3.38E-04–4.21E-02 (11)	EGF signaling	1.97E-03 (4)
	Gene expression	3.99E-04–2.13E-02 (5)	VEGF signaling	9.31E-04 (4)
	Cellular growth and proliferation	4.93E-04–4.83E-02 (19)	Thrombopoietin signaling	1.38E-03 (5)
	Cellular assembly and organization	1.11E-03–4.21E-02 (20)	IL-2 signaling	1.69E-03 (4)
nLDL 5h	Cell cycle	2.10E-06–4.89E-02 (32)	PPARα/RXRα	8.83E-05 (13)
	Cellular development	1.98E-04–4.45E-02 (28)	Activation TGF-β signaling	1.34E-04 (9)
	Cellular movement	6.91E-04–4.35E-02 (19)	Molecular mechanisms of cancer	2.58E-04 (20)
	Cell-To-Cell signaling and interaction	9.18E-04–2.63E-02 (10)	Regulation of IL-2 expression in activated and anergic T	7.06E-04 (8)
	Cellular growth and proliferation	1.34E-03–4.76E-02 (34)	Lymphocytes, VEGF signaling	9.2E-04 (8)
nLDL 24h	Cellular movement	7.09E-05–4.16E-02 (8)	LXR/RXR activation	2.33E-03 (4)
	Gene expression	9.24E-05–4.97E-02 (21)	Polyamine regulation in colon cancer	6.93E-03 (2)
	Cell-To-Cell signaling and interaction	1.37E-04–5.00E-02 (21)	Oncostatin M signaling	2.81E-02 (2)
	Cell cycle	1.45E-04–4.97E-02 (14)	Germ Cell-Sertoli cell junction signaling	3.02E-02 (4)
	Cellular growth and proliferation	2.31E-04–4.97E-02 (31)	Production of nitric oxide and reactive oxygen species in macrophages	23E-02 (4)
oxLDL 1h	Cellular growth and proliferation	5.69E-07–4.74E-02 (34)	Corticotropin releasing hormone signaling	2.68E-03 (5)
	Cell cycle	1.00E-06–4.74E-02 (15)	Antiproliferative role of somatostatin receptor	2.82E-03 (4)
	Cellular assembly and organization	9.25E-05–4.74E-02 (11)	Tight junction signaling	2.89E-03 (7)
	Cellular movement	1.53E-04–3.81E-02 (22)	Endothelin-1 signaling	4.14E-03 (6)
	DNA replication,recombination and repair	2.76E-04–4.74E-02 (14)	Nitric oxide signaling in the cardiovascular system	4.88E-03 (4)
oxLDL 5h	Cell cycle	2.96E-06–4.46E-02 (32)	Endothelin-1 signaling	1.96E-03 (10)
	Cellular growth and proliferation	1.42E-05–4.22E-02 (57)	ILK signaling	2.36E-03 (10)
	Cellular movement	1.20E-04–4.66E-02 (48)	Relaxin signaling	3.01E-03 (8)
	DNA replication, recombination and repair	1.24E-04–4.46E-02 (11)	Role of macrophages, fibroblasts and endothelial cells in rheumatoid arthritis	3.11E-03 (14)
	Cellular assembly and organization	4.52E-04–4.22E-02 (18)	Mitotic roles of Polo-like kinase	5.27E-03 (5)
oxLDL 24h	Cell Cycle	1.04E-04–3.81E-02 (56)	RhoA signaling	1.22E-02 (9)
	Cellular movement	1.55E-04–3.81E-02 (56)	Mitotic roles of Polo-like kinase	1.49E-02 (6)
	Cellular development	5.43E-04–3.81E-02 (58)	Germ cell-Sertoli cell Junction signaling	1.71E-02 (12)
	Cellular growth and proliferation	6.93E-04–3.81E-02 (92)	Polyamine regulation in colon cancer	1.77E-02 (3)
	Gene Expression	8.45E-04–3.81E-02 (26)	Leukocyte extravasation signaling	2.59E-02 (12)

Both lists were based on the Ingenuity Pathway Analysis (IPA).

*P values correspond to a range between the highest and the lowest significant result.

Our data show at least 9 well-differentiated molecular phenomena related to the physiology of vascular smooth muscle cells where the intensity and fate of cell responses depend on the exposure time to the stimulus (Table 2). Hence, primarily our analysis is focused on highlighting the contribution of those genes showing significant changes in order to integrate the molecular phenomena involved in vascular smooth muscle cells during a short and a long-term oxidative stimuli.

**Table 2:** hVSMC showing differential time dependent (1 to 24h) gene expression when exposed to nLDL or oxLDL.

Gene symbol	Gene description	Fold change*						Location
		nLDL 1h	nLDL5h	nLDL 24h	oxLDL 1h	oxLDL 5h	oxLDL 24h	
FOS	FBJ Murine Osteosarcoma Viral Oncogene Homolog	**4.147**	1.154	1.714	1.346	1.119	1.176	Nuclear, transcription factor
EGR1	Early Growth Response 1	**2.938**	-1.667	-1.118	-1.044	-1.814	-1.638	Nuclear, transcriptional regulator
DUSP1	Dual SpecificityPhosphatase 1	**2.157**	1.359	**2.102**	-1.055	-1.055	-1.009	Nuclear
MIR21	MicroRNA 21	**2.073**	1.254	**2.191**	-1.475	1.326	1.903	Cytoplasm
MMP16	Matrix Metallopeptidase 16	**-3.670**	**-3.716**	1.779	1.044	1.264	1.856	Cell membrane, Extracellular side
PHF3	PHD finger protein 3	**-3.109**	-1.458	1.788	-1.088	**2.043**	**2.428**	Nucleus
EXOC5	Exocyst Complex Component 5	**-3.094**	-1.203	-1.086	1.153	-1.025	-1.034	Cytoplasm
RB1CC1	RB1-Inducible Coiled-Coil 1	**-2.775**	-1.888	1.863	1.048	1.824	**2.499**	Nucleus, Cytoplasm, Cytosol
DST	Dystonin	**-2.771**	**-2.370**	1.355	-1.029	1.722	**2.180**	Cytoplasm, cytoskeleton
PPP1R10	Protein Phosphatase 1, Regulatory Subunit 10	1.032	**3.601**	**2.723**	-1.080	1.579	**2.996**	Nucleus
HIST1H2BF	Histone Cluster 1, H2bf	1.365	**2.348**	1.009	1.079	-1.114	-1.102	Nucleus, Chromosome
DLX5	Distal-Less Homeobox 5	1.273	2.070	**2.490**	1.054	**2.216**	1.887	Nucleus (by similarity) Membrane; Multi-pass membrane protein
SLC7A11	Solute Carrier Family 7 Member 11	**-2.692**	**-4.549**	1.872	1.040	1.572	**3.214**	Plasma membrane
TIPARP	TCDD-Inducible Poly (ADP-Ribose) Polymerase	-1.186	**-3.888**	**-3.101**	-1.521	**-2.713**	**-2.868**	Nucleus
ROCK2	Rho-Associated, Coiled-Coil Containing Protein Kinase 2	**-2.495**	**-3.635**	1.388	1.053	1.177	1.910	Cytoplasm, cytoskeleton, Cell membrane, nucleus
GLS	Glutaminase	**-2.001**	**-3.522**	-1.155	1.018	-1.289	1.024	
DDIT3	DNA-Damage-Inducible Transcript 3	1.090	1.384	**4.856**	-1.277	**2.378**	**2.370**	Cytoplasm, Nucleus
ABCA1	ATP-Binding Cassette, Sub-Family A (ABC1), Member 1	-1.346	-1.550	**4.367**	-1.195	1.909	**4.867**	Membrane
OGN	Osteoglycin	-1.741	-1.166	**3.228**	-1.026	1.354	**2.438**	Secreted, extracellular space, ECM
MYC	V-MycMyelocytomatosis Viral Oncogene hom	1.044	1.620	**3.180**	1.037	**2.286**	**2.891**	Nucleus
HSD17B6	Hydroxysteroid (17-Beta) Dehydrogenase 6	1.042	-1.293	**3.033**	-1.068	**2.525**	**3.773**	Microsome membrane, Lumenal side, Early endosome membrane
INSIG1	Insulin Induced Gene 1	-1.677	-1.381	**-4.189**	1.520	1.487	-1.456	Endoplasmic reticulum membrane
LDLR	Low Density Lipoprotein Receptor	1.124	-1.100	**-4.103**	-1.017	1.387	-1.375	Cell membrane
KIAA1199	KIAA1199: Colon Cancer Secreted Protein 1	-1.115	-1.318	**-3.627**	-1.152	-1.368	**-3.831**	Cytoplasm
ID3	Inhibitor of DNA Binding 3, Dominant Negative HLH-P	1.078	**-3.113**	**-3.244**	-1.227	**-3.767**	**-3.848**	Nucleus
IL1R1	Interleukin 1 Receptor, Type I	-1.434	**-3.118**	**-3.232**	-1.144	-1.660	-1.849	Membrane
CALCRL	Calcitonin Receptor-Like	-1.459	-1.361	**2.506**	**3.426**	**3.510**	**5.036**	Cell membrane
MYOZ2	Myozenin 2	1.001	-1.134	1.067	**3.125**	**2.142**	**3.038**	Cytoplasm, myofibril, sarcomere
GUCY1A2	GuanylateCyclase 1, Soluble, Alpha 2	-1.037	-1.101	1.499	**3.031**	**2.964**	**3.819**	Cytoplasm
TNFSF4	TNF (Ligand) Superfamily, Member 4	-1.195	1.032	1.757	**2.930**	**4.342**	**3.800**	Secreted
TFPI2	Tissue Factor Pathway Inhibitor 2	-1.382	-1.329	1.109	**-4.308**	**-2.831**	**-3.133**	Nucleus, Cytoplasm
KIAA0101	PCNA-binding protein	-1.385	-1.059	-1.438	**-4.219**	**-4.671**	**-5.105**	Nucleus, cytoplasm, perinuclear region
PTGS1	Prostaglandin-Endoperoxide Synthase 1	1.045	1.052	-1.131	**-4.067**	**-3.884**	**-3.130**	Endoplasmic reticulum and Microsome membrane
BEX1	Brain Expressed, X-Linked 1	1.107	-1.051	-1.126	**-3.743**	**-4.095**	**-3.258**	Nucleus, Cytoplasm
ACTG2	Actin, Gamma 2, Smooth Muscle, Enteric	1.261	1.274	-2.802	**-3.727**	**-3.614**	**-7.261**	Cytoplasm
IL33	Interleukin 33	-1.110	1.142	1.467	**2.685**	**4.110**	**3.141**	Nucleus, cytoplasmic vesicle
PPM1K	Protein Phosphatase, Mg2+/Mn2+ Dependent, 1K	-1.409	-1.579	1.441	1.005	**3.570**	**3.080**	Mitochondrion matrix
RASSF9	Ras Association (RalGDS/AF-6) Domain Family (N-terminal) Member 9	-1.008	1.462	**2.080**	-1.085	**3.546**	**2.991**	Accumulates on perinuclear endosomes
TEK	Tyrosine Kinase, Endothelial	-1.268	-1.047	**2.220**	1.072	**2.154**	**4.193**	Cell membrane, cytoplasm, secreted
CMAHP	CytidineMonophospho-N-Acetylneuraminic Acid Hydroxylase, Pseudogene	-1.512	-1.242	2.440	1.285	1.917	**3.892**	Cytoplasm (infered)
OCLN	Occludin	-1.030	1.006	1.412	-1.181	**2.889**	**-4.671**	Membrane; Multi-pass membrane protein

* Genes showing a significant expression change are indicated with bold numbers (P<0.05, FC>2.0).

TNF, Tumor Necrosis Factor; HLH-P, Helix-Loop-Helix Protein; ECM, extracellular matrix

#### Redox Balance

It is well-known that an oxidative stress condition generated by reactive oxygen species (ROS) promotes cardiovascular disease by damaging molecules such as DNA, RNA, carbohydrates, lipids and proteins. Nevertheless, the precise mechanism by which they deteriorate vascular function and promote vascular remodeling *in vivo* has yet to be elucidated. To date, it is known that during early stages of atherosclerosis, cell proliferation is stimulated, while at late stages, VSMC promote apoptosis and therefore plaque instability[29].

On the other hand, the best identified enzymatic pathways exclusive to eliminate ROS are mediated by catalases and a variety of peroxidases, transferases, reductases, oxidoreductases, peroxiredoxins and superoxide dismutases (SOD) [8, 30]. From our list of genes and proteins considered by the Ingenuity Pathways Analysis (IPA) associated with an oxidative stress response and the maintaining of a redox state, we found that catalase (CAT) seems to carry out the main role in detoxification, and therefore proposed as a therapeutic target against the adverse effects of ROS associated to cell dysfunction and damage over molecules such as lipids, DNA, RNA, carbohydrates and proteins.

It is evident that VSMC incubated for long periods of time with nLDL present a transcriptomic response similar to the one seen at shorter times when oxLDL are employed. This series of experiments are consistent with the concept that metabolically oxidized lipids and modified proteins evolve with time, where short time incubation of cells in the presence of artificially modified oxLDL seem to emulate the cellular response carried out by hVSMC when incubated for longer periods of time in the presence of non treated nLDL. Therefore, we believe this approach might be a good model to reproduce and study the molecular events that might be happening *in vivo* with time during the process of atherogenesis.

#### Apoptosis and Cell Cycle Related Genes

The control of the cell cycle proved to be an essential regulation process when hVSMC were exposed to LDL (Table 2). For gene ontology (GO) categories enriched in the IPA analysis, we observe an over-representation of genes associated with the cell cycle in virtually all our experimental conditions. At early exposure times employing nLDL or oxLDL, genes FOS and EGR1(Early Growth Response 1) are upregulated with a fold change above or below the threshold of ±2 (4.1 and 2.9, respectively). These genes are indicators that the regulation of cell proliferation, differentiation and transformation is taking place, and agree with previous observations reported during early stages of atherosclerosis, where ROS stimulate VSMC growth [30]. However, at exposure times of 5h onward, both FOS and EGR1 drastically decrease their expression, thus indicating that it is only at short periods of lipoprotein exposure that their activity seems to be regulated.

During the regulation of the cell cycle, two main trends seem to be present; nLDL exposure increases ATM expression and decreases PLK1, a member of the Polo-like kinases (PLKs) family. Since these genes regulate changes in mitosis, cytokinesis and the response to DNA damage [31, 32], it seems they determine the onset for the cell cycle arrest. At this very early condition, FOS expression is increased at 1 h exposure with both native and oxidized lipoproteins (4.1 and 2.9respectively), while at late periods of time (24 h) MYC increases with both stimuli (3.18 and 2.89). Studies where cells were stimulated using fetal bovine serum show two waves of an increased expression of FOS. The first one at 7.5 minutes, when protein is found in the endoplasmic reticulum; and the second at 20 min with a maximal peak at 1h, when the protein is found in the nucleus. These observations are supported by findings showing that c-Myc expression is strongly regulated through cell cycle control and that dysregulation can explain the shortening of G1 and the progression of G1/S [33, 34].

On the other hand, the increase in the expression of several genes such as DLX5, a transcription factor involved in bone development and essential for the differentiation of osteoblasts promoting cell proliferation, perfectly correlates with the increase in MYC expression. It has been reported that DLX5 upregulates the activity of the MYC promoter [35], which presents a strong transcriptional repression effect for genes associated with cell cycle arrest, like GAS1, p15, p21, p27, and GADD34, -45, and -153[36].

In the presence of oxLDL, genes like APC are upregulated (2.2 and 2.3 oxLDL 5h and oxLDL 24h, respectively), showing a tendency to promote the process of apoptosis, transcriptional activation and cell migration. APC seems to act along with DDIT3 (4.8 and 2.3 nLDL 24h, oxLDL 24h), also associated with stress-activated apoptosis in the endoplasmic reticulum. Both regulate the inflammatory response induced by activation of caspase 1 (CASP1) and interleukin-1 beta (IL1B). Thus, the dynamics among mitogenic signals, cell cycle arrest, migration, apoptosis, DNA repair, and other cell and molecular functions is affected depending on the stimulus received and the time of exposure as seen in Table 2.

The levels of expression observed in all formerly mentioned genes are congruent with a system trying to avoid cell arrest. Moreover, we observed regulation of genes such as CYR61, which favors cell proliferation and play an important role in repair by regulating the expression of genes involved in angiogenesis, inflammation and remodeling of extracellular matrix[37]. To date, it is known that during the early stages of atherosclerosis, cell proliferation is stimulated, while at late stages, VSMC promote apoptosis and therefore plaque instability[30]. Our results suggest that hVSMC proliferate well after 24 hours exposure with nLDL, while exposures to oxLDL seems to trigger cell migration and cycle arrest. Together, these last effects could constitute a rather dangerous response in the formation of an atheroma. Although a low oxidative stress level due to naturally modified LDL might eventually start or increase the size of incipient atheromatous plaques, a chronic exposure to these modified lipoproteins that could with time be extensively oxidized will promote cell migration, cell-cycle arrest and would lead to a full developed atherogenic event.

#### Remodeling of the extracellular matrix

Cell exposure to oxLDL remodels the extracellular matrix as indicated by the overexpression of the metalloprotease MMP16 (MT3-MMP), with the largest effect found at 24 h (Fold change 3.1), and the associated downregulation of TIMP1, a natural inhibitor of metalloproteases. In close relationship to this remodeling event, endocytosis associated with the phagocytic route for proteins, extracellular matrix degradation[38]and cell movement has been reported[39]. In the same context, genes like ICAM1 (CD54) involved in cell adhesion are downregulated, indicating an increase in cellular migration and delocalization, phenomenon that has been observed *in vivo* in the atherosclerotic plaque. Likewise, integrins 3 and 5 (ITGA3 and ITGA5) also decrease their expression when they receive signals associated to a large generation of ROS.

#### DNA Repair

The IPA database reports 330 genes associated with DNA repair. In the presence of oxLDL we found a significant representation of several of these genes within the top 5 overexpressed molecular and cellular functions. For example, 3, 5 and 11 genes were overexpressed above the +2 threshold at 1, 5 and 24 hours, respectively. The overexpression observed at 24 hours of genes such as KIT (2.6), XRCC4 (2.05), ERCC4 (2.14) and ERCC6 (2.06), among others, suggest a response attempting to maintain cellular integrity, even when damage caused from early incubation times with oxLDL had started. Together, these changes affect the final equilibrium state between an increase in apoptotic or cell maintenance signals.

#### Cholesterol Efflux

In general, under our experimental conditions we observed that only a few genes related to the metabolism of cholesterol were significantly regulated. Nevertheless, it is important to stress the remarkable overexpression of the cassette transporter ABCA1 (fold change 4.8), a critical transporter contributing to cell cholesterol efflux. We also observed other members of the A and C families importantly upregulated, such as ABCA6, ABCA8, ABCA9 and ABCC9 with fold changes 2.9, 2.05, 3.37 and 3.33, respectively. Among these transporters, only ABCA9 and ABCA6 have been reported to be upregulated in the process of differentiation from monocytes to macrophages and downregulated in the process of cholesterol internalization[40, 41].

#### Endocytic Mechanisms

**Fig 2:**
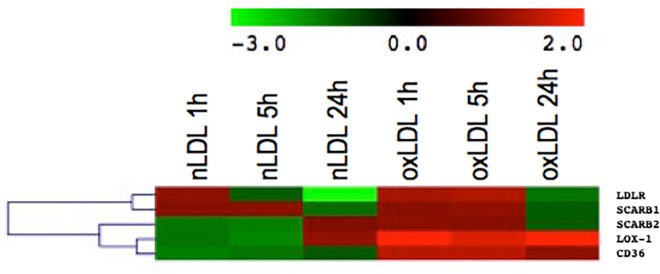
Hierarchical cluster analysis of several receptors and their expression when the six experimental conditions used (nLDL 1h, nLDL 5h, nLDL 24h, oxLDL 1h, oxLDL 5h and oxLDL 24h) are compared to parental hVSMC cells.

The behavior shown by the expression of receptors associated with the internalization of both types of LDL (native and oxidized) corresponded as expected. The oxLDL receptor (OLR1) and the CD36 receptor (SCARB3) were overexpressed when cells were treated with oxLDL, and considerably downregulated the expression of the LDL receptor (LDLR) at long periods of time when exposed to nLDL(-4.1). Meanwhile, the expression of the scavenger B1 and B2 receptors was turned on at short periods of incubation with oxLDL (Fig 2), indicating the dynamics associated with the type and degree of oxidation, data that strongly agree with the known variability effect of oxLDL reported in literature [42].

#### Calcium homeostasis

In a similar fashion to the pattern observed with other genes, hVSMC treated for a short time with oxLDL or for longer periods of time using nLDL, increase the expression of SPP1, BMP6 and CALCRL corresponding to genes that promote the differentiation and maturation of osteogenic cells and whose activity may produce pathologic biomineralization in vascular tissues. Additionally, we observed that oxLDL decreases TAGLN and ACTA2 expression, two gene markers for the VSMC phenotype. Taken together, these data suggest a transdifferentiation step similar to that observed in VSMC exposed to high glucose concentrations, showing these cells an interesting plasticity depending on the stimulus they receive [8].

On the other hand, although VSMC do not naturally express proteins related to calcification, our group has found that under an atherogenic condition they are capable of expressing SPP1[43], a protein that regulates the formation of hydroxyapatite crystals. To our knowledge, this is the first report related to osteogenic signaling by bone morphogenetic proteins (BMP) when exposed to oxLDL.

#### Membrane Trafficking and Exosome

As a consequence of ROS increase, genes like KIF4A and PRC1 associated with the movement of membranous organelles, intracellular transport and cytokinesis are downregulated. This finding is in agreement with the tendency towards cell cycle arrest and the increase in the expression of INHBA and CALCRL, suppressants of the proliferation process. Likewise, genes such as RAB33B which is overexpressed, might be important in defining the steps of vesicular transport along several endocytic routes, and suggests how the dynamics of cargo systems and the formation of the autophagosome may work[44]. It has been suggested that this mechanism involves the degradation of cytoplasmic components in response to the initiation of macro-autophagia [45].

#### Immune Response and Inflammation

**Fig 3:**
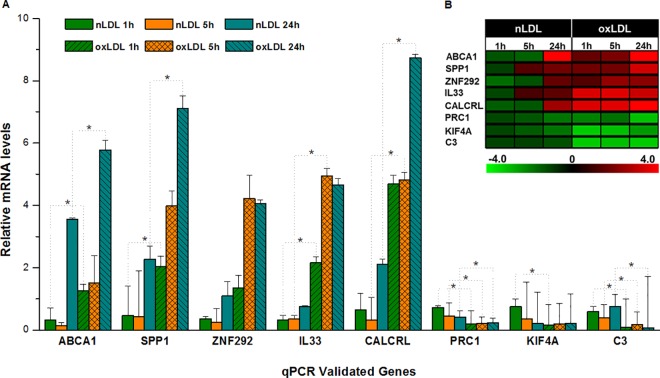
Validation of microarray results by quantitative RT-PCR (A) Gene expression of ABCA1, SPP1, ZNF292,IL33, CALCRL, PRC1, KIF4A and C3 determined by quantitative real-time RT-PCR. Bars represent means ± SD (n = 3). Relative gene expression levels, normalized to GAPDH expression. Values superscribed by asterisks (*) are significantly different from nLDL and oxLDL groups (B) Heatmap Expression profile (microarray data).

The inflammatory process shows important changes in hVSMC marker genes during exposure to oxLDL, strengthening their relationship with the process of oxidative stress. SPP1, which is recognized as a proinflammatory cytokine is upregulated along with CALCRL, regulation that shows not only to be significant at all incubation times when exposed to oxLDL, but also to be important when cells were incubated for a long period of time with nLDL (Fig 3). In this context, it is noteworthy the overexpression of IL33 when cells are exposed to oxLDL since this molecule has been shown to present a protective effect during the inflammation process of adipose tissue in mice by inducing the production of Th2 cytokines such as IL-5, IL-13, IL-10 [46].

On the other hand, cells exposed for 24 h with nLDL show a significant regulation upon defense and communication processes associated to the immune system, suggesting that CAD and complications due to an inflammatory process start from the exposure at short times with nLDL. Since the regulation of these processes by key molecules such as IL33 permits the restitution of a normal expression, this modulating process might also become a potential therapeutic target.

On the other hand, we have observed that nLDL unlike oxLDL regulate innate immunity processes and the communication between cells, even though the immune system only recognizes the latter as an antigenic stimulus. Therefore, it is possible to think that complications due to inflammation at the vascular level seen in CAD might start at the early stages of the atherogenic process. Therefore, modulation of these inflammatory processes by key molecules such asIL33, permits the restitution of gene expression close to a normal stage and in consequence considered as a protective molecule.

#### Validation of Microarray Data by Quantitative PCR

The validation of our transcriptomic expression analysis represent genes involved in the control of the diverse functions carried out by hVSMC. Five of the clearly upregulated genes in response to an oxidative stress condition included ABCA1, SPP1, ZNF292, IL33 and CALCRL, while PRC1, KIF4A and C3 were downregulated. Fig 3 shows the correlation data between data obtained from the microarray and changes in gene expression determined by qPCR using Taqman probes.

### Identification of transcriptional regulation

#### Exposure to native LDL for 1 hour (nLDL 1h)

Using the tools for the identification of transcriptional regulation cascades implemented IPA Upstream Analysis, it is predicted that receptor CD24 is inhibited. This prediction is based on 11 genes reported to be regulated by this receptor (VPS13C, USO1, TTC3, TPR, SCAF11, RANBP2, NRIP1, NIPBL, JMJD1C, EDEM3 y DNAJC13) with fold changes > 2 defined as the cut-off value. These genes are mainly associated with signaling routes like RAN, protein degradation by ubiquitination and vesicle transport, indicating as expected, that LDL turn on the processes of nucleus-cytoplasm transport and membrane recycling. The role of CD24 should be noticed since this receptor is generally found on the surface of endothelial cells, where it recognizes among other ligands, P-selectin which is associated to the process of rolling and tethering, essential steps in leukocyte recruitment and diapedesis [47].

#### Exposure to native LDL for 5 hours (nLDL 5h)

The upstream analysis at 5h with nLDL shows the predicted inhibition of 6 genes (CD24, SYVN1, MYC, PDGF BB, IFNA2 and EIF2AK2) and their associated signaling cascades involving 53 genes. From these, seven downregulated genes stand out since they show a consistent inhibition of MYC expression which, on the other hand, at larger incubation times would increase their expression at similar levels between native and oxidized LDL. Likewise, the regulation of the importin- exportin-mediated nucleus-cytoplasm transport systems, like XPO1 (Fold change-2.9) are downregulated. While 12 genes associated with SYVN1 inhibition, some of them belonging to different protein carrier families such as SLC7A5, SLC39A10, SLC30A1, are downregulated along with genes such as RAB10 from the RAS family. The predicted inhibition of platelet-derived growth factor BB (PDGF BB) could be related to VSMC proliferation and migration inhibition through AKT phosphorylation mechanisms[48].Only one transcriptional regulation cascade is affected with 6 downregulated genes, predicting the activation of collagen 18A1 (COL18A1).

#### Exposure to native LDL for 24 hours (nLDL24h)

Along this condition, 12 genes predict the inhibition of signaling cascades associated with NFkB and IL13, congruent with ROS formation considering that 24h incubation produces a moderately oxidized LDL (MM-LDL). ROS lead to a possible inhibition of the NFkB route, which would generate an anti-inflammatory process as previously associated with the oxidation state of LDL[49, 50].

#### Exposure to oxidized LDL for 1 hour (oxLDL 1h)

The downregulated expression of SEMA7A, PTGS1, MT2A and HLA-B genes predict the inhibition of the signaling cascade associated with TGM2 and the activation of the methylation processes. Five downregulated genes (TOP2A, PBK, FJX1, DLGAP5 and BUB1B) associated with KDM5B predict their activation, being DLGAP5 a regulator of integrity of tight junctions, and BUB1B an essential component of the mitotic checkpoint.

#### Exposure to oxidized LDL for 5 hour (oxLDL5h)

10 genes with upregulated and downregulated expression at fold changes between±2 support the inhibitory prediction carried out by ROS of IL13 and ERK, as an indicator of anti-inflammatory signaling control and of transcriptional control. These results are consistent with the levels of expression of genes induced by nLDL at 24 hours.

#### Exposure to oxidized LDL for 24 hour (oxLDL24h)

**Fig 4:**
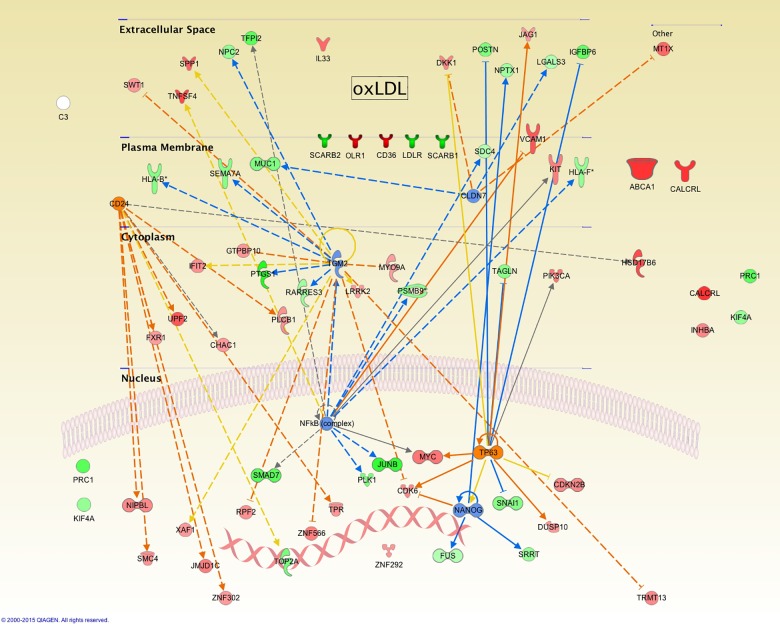
Summary of main changes observed in gene expression and functions of hVSMC as a result of exposition to oxLDLs. Genes found over- or under-expressed in comparison to a control condition are shown (red and green respectively), and the predicted activation or inhibition in orange and blue.

Here, 43 genes predict the inhibition of 4 genes and the activation of 2 more along with their associated signaling cascades. Fig 4 depicts a representation based on the cellular location according to Gene Ontology (GO) and the known interactions with other biomolecules for each one of the affected genes, showing a mechanistic representation of possible actions performed by these 6 genes. Hence, we can conclude that at longer exposure times and greater oxidative stress, cellular controls are mainly affected in relation to the inhibition predicted for NANOG, as an indicator of the regulation for cellular pluripotentiality, supporting the idea of transdifferentiation. Additionally, the inhibition of Claudin 7 (CLDN7) as a protein forming part of tight junctions would correlate with the loss of cell architecture. This inhibition correlates with the expression of MUC1, MT1X, LGALS3 and DKK1 genes.

Under these conditions, eight genes predict the activation of CD24, a phenomenon opposite to what we observed with nLDL at early periods of time, where its inhibition was predicted. This phenomenon not previously reported for this cellular system could modulate activities such as cell migration. Interestingly, the activation of TP63 and the translated protein p63, member of the p53 transcription factor family, would be an indicator for the preservation of the proliferative potential of these cells as seen in the auto-renewal of limbal stem cells regulating their mitogenic rate, phenomenon also observed during corneal repair[50].

Finally, in order to support our finding correlating effects observed with cells between long incubation times employing nLDL particles and short incubation times using oxLDL, the degree of oxidation present in the culture media at the different conditions employed was directly measured.

**Fig 5:**
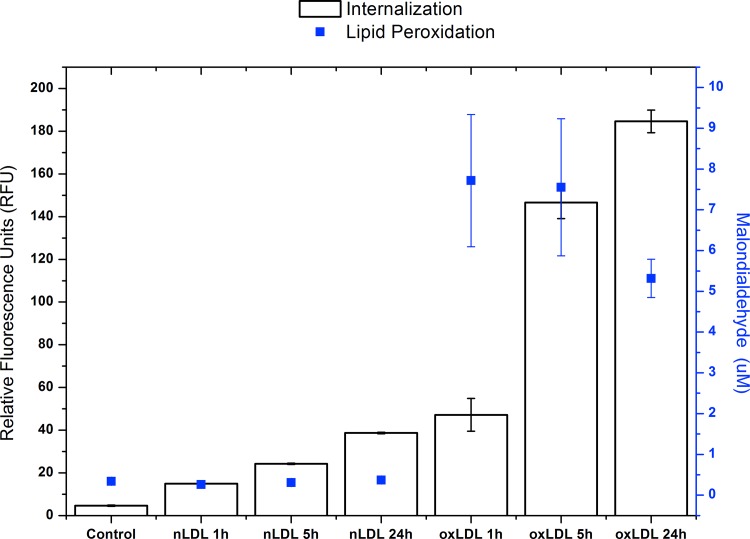
Internalization Assays and lipid peroxidation in VSMC cells. White boxes corresponding at internalization in VSMC cells of 7.5 μg/mL of nLDLs or oxLDLs DiI label for 0 (Control), 1, 5, and 24 h under medium minimal culture conditions. The magnitude of internalization shown on the left side in the y-axis as relative fluorescence units (RFU) at 530/590 nm (excitation/emission). The blue squares correspond to the level of peroxidation of media supernatant after the same conditions. The lipid peroxidation level is shown in the y-axis on the right side expressed as [μM] of malondialdehyde.

Fig 5 clearly shows that while culture media containing oxLDL present important malondialdehyde values that tend to decrease with time in parallel to the process of lipoprotein internalization through scavenger receptors, along with time, nLDL show a modest tendency to be mildly oxidized and therefore probably still being recognized and internalized through the LDL receptor as previously suggested.

## Discussion

Since oxygen is known to be the key element in the preservation of life as we know it, the presence of free oxygen radicals in circulation is a reality due to the many oxidative reactions of the cell. Along the respiratory metabolism and under specific conditions, cells convert oxygen molecules to highly reactive oxygen species by losing electrons and becoming highly efficient radicals that affect nucleic acids, carbohydrates, lipids and proteins. For years now, these reactive oxygen species have been associated with the process of atherogenesis, where a vascular inflammatory condition together with the chemical modification of lipoproteins and endothelial damage, develop atherosclerosis and ultimately cardiovascular disease.

Taking into account the complicated mechanisms and signals involved in this process, many laboratories have focused their research not only trying to find a way to overcome the deleterious effects of free oxygen radicals, but also working in the search for gene-based markers through transcriptomics and metabolomics that might help to recognize when oxidative stress has reached a dangerous level. Although there is a published microarray that presents the response of oxLDL upon human coronary artery smooth cells employing incubation times starting at 3h[51], according to our study using a starting incubation time of 1h, important differences between the two studies are found. A period as short as 1h permitted us to observe changes among others associated to the expression of microRNAs and matrix metallopeptidases. Another relevant difference between the two studies is related to the intrinsic properties associated to the phenotypes of cells used as experimental model. Coronary smooth cells show a highly differentiated phenotype even after been cultured with growth factors added. This cell type seems to be less responsive when exposed to different stimuli and also exhibits a lower synthesis activity[52, 53]. In contrast, hVSMC used in this study are not terminally differentiated and therefore plastic enough to respond showing a contractile phenotype after a physiological stress, or a synthetic phenotype when physical (mechanical) stimulation is used[54, 55]. In this sense, VSMC isolated from hypertensive individuals show a significant increase in ploidy and hypertrophy[56]. Also, Angiotensin II involved in the development of cell contraction and eventually of hypertension, regulates polyploidization in this cell type as well as the hepatocyte through the Akt1 pathway[57]. Moreover, since both smooth muscle cell types express different sets of genes that are down or up-regulated in response to oxidative stress, it is not difficult to think that accordingly to their position in the media layer of the artery wall, these cells might respond differently to different kinds of stimuli, phenomena most surely important in the progression of the atherosclerotic plaque *in vivo*.

Our work contributes with the exploration of such gene-based markers when human VSMC have been exposed to different levels of oxidated LDL particles, employing both native LDL that naturally become oxidized with time during incubation, and chemically oxidized LDL.

Data obtained from our assays searching for differential expression patterns caused by nLDL and oxLDL contribute to understand how cell systems such as hVSMC respond to harmful stimuli aiming at achieving cell survival within a favorable energetic landscape.

Genes regulated by nLDL are involved in a wide spectrum of biological processes suggesting redundancy of genes regulated by oxLDL that in turn are mainly focused prioritizing the reestablishment of key cell survival functions. Among these functions, it can be mentioned gene transcription and its involvement in promoting adaptation into the environment; DNA protection from possible damage carried out by ROS, and cell cycle control as a response to a hostile environment.

Therefore, according to our analysis, cell cycle control is one of the most important processes affected, showing a strong regulation by oxLDL and nLDL. Interestingly, several reports have described a close relationship with cell growth arrest between the up-regulation of ATM, APC, DLX5 and DDIT3 and down-regulation of PLK1, PRC1 and KIF4A. These genes have shown similar changes at short exposure times with oxLDL to those observed at long-term exposure times with nLDL. Although several reports suggest that oxLDL exposure might stimulate proliferation[56], our results show that under high oxidative stress levels and the presence of oxLDL, a transcriptomic pattern seems to predispose cells to arrest growth, stop proliferation and prevent apoptosis. Moreover, when cells are briefly exposed to nLDL, cell growth arrest is avoided and proliferation seems to be stimulated, results congruent with studies where a moderate oxidative stress level was used[58]. Therefore, the type of response shown by cells drastically varies according to the kind and degree of oxidation LDL particles might present. In this sense, the extent of the natural process of lipoprotein modification found in an *in vivo* situation as an example as to what it might be occurring in the atheroma, could be determinant in the progression of the lesion.

For some time now, there has been controversy as to the meaning of minimally modified LDL. It has been established that lipoprotein particles need to be sufficiently modified in order to be differentiated from unmodified LDL that maintain their ability to bind to the LDL receptor and therefore still not recognizable by scavenger receptors. In this sense, as shown in the present study a modification of nLDL achieved with time that corresponds to the mildest exposure condition observed with oxLDL might be sufficient for an important induction of a series of gene responses. The transcriptional behavior of hVSMC in culture at 24 hours exposition with nLDL corresponds to the response observed at initial times in the presence of oxLDL. It is likely that slight differences between moderately and extensively oxidized LDL particles lead to opposite transcriptional responses. A modest oxidative stress due to long term naturally oxidized LDL particles seem to promote cell proliferation as expected on the first stages of atherogenesis, while a greater oxidative stress from a short term exposure with chemically treated particles apparently promote cell growth arrest[58, 59].Since it is commonly accepted that the process of LDL oxidation in plasma is not a common reaction due to the presence of antioxidant molecules such ascorbate and tocopherol, oxidation mainly takes place in the subendothelial space where the presence of antioxidant molecules is much less abundant[60]. Therefore, this phenomenon most probably is associated to the possibility that naturally modified LDL particles presenting different degrees of oxidation at the different stages of the atherosclerotic lesion, might be sending differentiated defense signals through the LDL receptor before such particles are recognized by the scavenger receptors in an advanced type of lesion.

Our analysis also shows a tight regulation of inflammation related processes, whose development has been shown to be of paramount importance from the genesis of fatty streaks up to the advanced stages of atherosclerosis[61, 62]. Cells exposed to nLDL and oxLDL overexpress characteristic cytokines of a proinflammatory process such as IL33 and SPP1. This cellular response generates an inflammatory process within the vessel through communication with the different cell lines of the immune system. It is also interesting to consider that several molecules involved in inflammation have been shown to present important functions in other biological processes, such as CALCRL and SPP1, which have been demonstrated to be intimately involved in osteogenic cell differentiation. The presence of cells with a phenotype favoring calcium deposits is of great relevance in the vessel physiopathology, affecting long term cell survival[7]. Our group has described an *in vitro* model investigating the inhibition of hydroxyapatite formation [Ca10(PO4)6(OH)2] as a consequence of the presence of SPP1[43]. Unlike its role in inflammation, SPP1 activity has been shown to be atheroprotective since an increased concentration of SPP1was detected in the arteries of rabbits exposed to high cholesterol diets and developing atherosclerotic lesions. Employing this experimental animal model and in spite of the presence of calcium deposits, the presence of SPP1 and its overexpression by VSMC corresponds to an oxidative stress stimuli, for instance, caused by oxLDL. On the other hand, downregulation of TAGLN and ACTA2, markers for a smooth muscle phenotype, would promote a poorly defined differentiation that in turn could allow a calcification-promoting phenotype.

The present work shows that the compound media layer of hVSMC produces a highly rich and versatile transcriptomic response triggered by the presence of chemically modified lipoproteins, supporting the concept that CAD is a condition consolidated by the integral response of the intima, media and the adventitia. This information shows that the initial response of cells towards a proatherogenic stimulus can be considered a functional defense against the process of atherogenesis, suggesting that the true atherogenic potential lies in a persistent aggression that exceeds cell defenses.

Gene expression of hVSMC in the presence of nLDL for less than 5 hours seems to promote the development of this functional defense metabolism; however, during longer incubation times, cells in the presence of nLDL might render these particles prone to oxidative modifications and the promotion of a proper oxidative stress condition. Although under these initial mild conditions, cell defense mechanisms respond mainly through proliferation, a long exposure to these conditions can be converted into a potentially proatherogenic stimulus. As a whole, from our results it is possible to propose that the presence of a low oxidative stress level can be considered a functional defense process. When a low oxidative stress level changes to an extreme one over time, the true atherogenic potential emerges.

## Methods

Human plasma samples were obtained from Blood Bank healthy donors at the National Medical Center “20 de Noviembre” that signed an informed consent. All protocols were performed according to the Declaration of Helsinki. To isolate LDL, plasma density was adjusted to a 1.019–1.063 g/ml by adding KBr and then, centrifuged at 360,000 x g for 8 h at 4°C. The top layer containing VLDL and IDL was discarded. LDL preparation was recovered and dialyzed against 150 mMNaCl, filtered through 0.45 μm filter and stored under nitrogen atmosphere at 4°C to reduce oxidation. LDL concentration was measured with the Micro BCA Protein Assay Kit (Pierce; Rockford, IL). Part of the concentrated LDL was reserved to use as native LDL (nLDL)[63–67].

To prepare oxLDL, 1 ml of nLDL (100 mg/ml) was incubated in a 10 μM CuSO4 solution in PBS without calcium, magnesium nor antioxidants at 37 Celsius degree for 20 h. The reaction was stopped with 100 μM EDTA and the preparation was dialyzed against 150 mM NaCl containing 240 μM EDTA (pH 8) and stored at 4°C until use[43, 64]. The oxLDL concentration was estimated reading in the UV range (215 nm) in a spectrophotometer UV/VIS Lambda 2S (Perkin-Elmer).

### Cell culture

The human vascular smooth muscle cells (VSMC) were originally obtained from the aorta of a healthy female patient. T/G HA-VSMC from ATCC (Cat. CRL-1999) were grown in 10 cm culture dishes with F12K medium containing 0.05 mg/ml ascorbic acid; 0.01 mg/ml insulin; 0.01 mg/ml transferrin; 10 ng/ml sodium selenite; 0.03 mg/ml Endothelial Cell Growth Supplement (ECGS); 10% fetal bovine serum (FBS), 10 mM HEPES, 10 mM TES, 100 μg/ml penicillin/streptomycin and 0.25 μg/ml fungizone. Cells were incubated in a humidified environment at 37 Celsius degree in an atmosphere of 95% air / 5% CO2.

### Internalization assays

VSMC in their fifth passage were starved for 1 h and internalization assays started by adding to the culture dish fresh medium containing 7.5 μg/mL of nLDLs or oxLDLs for 1, 5, and 24 h under standard culture conditions. For each experimental condition, 9 culture dishes were used and pooled dishes containing three biological replicates examined. For internalization assays, lipoproteins were labeled with 1,1´-dioctadecyl-3,3,3´3´-tetramethylindocarbocyanine perchlorate (DiI) (Molecular Probes, Eugene, OR, USA) [67], and cells incubated in the presence of Dil-labeled lipoproteins employing 96-well plates containing 20 x 10^3^ cells/well. After the internalization assay, cells were washed 5x with 0.2 mg/ml BSA (in PBS) and 2x with PBS, and fluorescence read in a Synergy HT Microplate Reader (Biotek Instruments, Inc., Winooski, VT) at 530/590 nm (excitation/emission). The oxidation level of supernatants obtained from the different pooled culture media in the presence of oxLDL or nLDL was estimated using the NWLSS Malondialdehyde assay according to the instructions provided by the manufacturer (Northwest Life Science Specialties, LLC, Vancouver, WA; Cat. NWK-MDA01), and read at 532 nm in a Lambda 2S spectrophotometer.

### In vitro transcription and hybridization to Affymetrix HumanGeneChip

Total RNA from 9 samples of VSMC in 3 different time courses (1, 5 and 24 hours) and two different treatments (nLDL = native LDL or oxLDL = oxidized LDL) were isolated with Trizol reagent (Invitrogen Life Technologies).

Samples were quantified using a NanoDrop ND-3300 spectrophotometer (NanoDrop Technologies) and RNA quality assessed using the RNA 6000 Nano Assay and analyzed using the Agilent 2100 Bioanalyzer (Agilent Technologies).

Total RNA was obtained from three culture plates exposed to the same treatment pooled with a yield of 200 ng of single pooled sample. Three technical replicates were prepared for each condition.

Target cDNA was prepared according to the Whole-Transcript (WT) Sense Target Labeling Protocol (Affymetrix). Briefly, 200 ng of pooled RNA was converted to one-strand cDNA using a Superscript II reverse transcriptase primed by a poly(T) oligomer. The second strand cDNA synthesis was followed by an in vitro transcription to generate cRNA.

cRNA products were used as a template for a second cycle of cDNA syhthesis where dUTPs incorporate to the new strand. cDNA was fragmented using uracil-DNA glycosilase and apurinapirymidin endonuclease. Fragments (40–70 mers) were then labeled by means of a biotin-labeled deoxynucleotide terminal addition reaction. The labeled cDNA product was heated to 95°C and hybridized to the Human Gene 1.0 ST microarray (Affymetrix) for 17 + 1 hr at 45°C. Samples were washed with low (SSPE) and high (100 mM MES, 0.1 M NaCl) stringency buffers and stained with streptavidin-phycoerythrin using the Affymetrix Fluidics Station 450 FS450_0007 protocol.

The GeneChip Scanner 3000 7G (Affymetrix) was used to collect fluorescent signals and the Expression Console software (Affymetrix) used to obtain intensity signal and quality data from scanned arrays. The software provides summary reports and metrics including average background, positive versus negative areas under the curve and expression ratios for the presence of spikes in controls, useful for array QC evaluation.

### q-PCR validation

To validate changes of down or up-regulated genes, transcript expression was analyzed by qPCR using the same RNA pools used in the Affymetrix arrays.

The cDNA was synthesized using the Taqman Gold RT-PCR kit (Life Technologies) from high quality RNA (RIN ≥ 9.8), according to the instructions provided by the manufacturer. All qPCR reactions were performed in triplicate using the Taqman Universal Master Mix and the indicated Taqman probes on a ViiA™ 7 real-time PCR system (Life Technologies) in a final volume of 20 μl. Temperature conditions and number of cycles were set as recommended by the provider. The quantitative analysis of transcripts was carried out using the comparative ΔΔCt method and GAPDH expression used as an endogenous control. A Welch Two Sample t-test was used and a P-value of <0.05 was considered statistically significant in all cases.

### Microarray Data Analysis

The Human Gene 1.0 ST is designed to investigate 28,869 well-annotated genes. Signal intensity from 21 arrays in.CEL format were analyzed using the Partek Genomic Suite version 6.4 (Partek). Raw intensity probes values were imported by setting up a robust multiarray analysis background correction. To identify differentially expressed genes, a two way ANOVA was performed. From the results of these analyses, genes with P < 0.05 and 2.0- fold change (FC) in either direction were identified as being differentially expressed.

Data presented and discussed in this study have been deposited in the NCBI's Gene Expression Omnibus3 and accessible through the GEO Series accession number GSE68021 (http://www.ncbi.nlm.nih.gov/geo/query/acc.cgi?acc=GSE68021).

### Network analysis

Network analysis, including the enrichment and upstream analysis was performed using the Pathway Analysis Suite (Ingenuity), where elaboration of pathways and networks was carried out.

## References

[1] The top 10 causes of death. Available from: http://www.who.int/mediacentre/factsheets/fs310/en/.

[2] INEGI (National Institute of Statistics and Geography) from: http://www.inegi.org.mx/est/contenidos/proyectos/registros/vitales/mortalidad/

[3] Lusis AJ. Atherosclerosis. Nature. 2000;407(6801):233–41. 10.1038/35025203 11001066 PMC2826222

[4] Libby P, Egan D, Skarlatos S. Roles of infectious agents in atherosclerosis and restenosis: an assessment of the evidence and need for future research. Circulation. 1997;96(11):4095–103. 10.1161/01.cir.96.11.4095 . 9403635

[5] Qi X, Zhang Y, Li J, Hou D, Xiang Y. Effect of PGC-1alpha on proliferation, migration, and transdifferentiation of rat vascular smooth muscle cells induced by high glucose. Journal of biomedicine & biotechnology. 2012;2012:756426 10.1155/2012/756426 22461724 PMC3303719

[6] Schachter M. Vascular smooth muscle cell migration, atherosclerosis, and calcium channel blockers. International journal of cardiology. 1997;62 Suppl 2:S85–90. 10.1016/s0167-5273(97)00245-3 . 9488199

[7] Schwartz SM. Perspectives series: cell adhesion in vascular biology. Smooth muscle migration in atherosclerosis and restenosis. Journal of Clinical Investigation. 1997;99(12):2814–6. 10.1172/JCI119472 9185501 PMC508129

[8] Stocker R, Keaney JF Jr. Role of oxidative modifications in atherosclerosis. Physiological reviews. 2004;84(4):1381–478. Epub 2004/09/24. 10.1152/physrev.00047.2003 . 15383655

[9] Katsuda S, Boyd HC, Fligner C, Ross R, Gown AM. Human atherosclerosis. III. Immunocytochemical analysis of the cell composition of lesions of young adults. Am J Pathol. 1992;140(4):907–14. 1562051 PMC1886367

[10] Stary HC, Chandler AB, Dinsmore RE, Fuster V, Glagov S, Insull W Jr., et al A definition of advanced types of atherosclerotic lesions and a histological classification of atherosclerosis. A report from the Committee on Vascular Lesions of the Council on Arteriosclerosis, American Heart Association. Arterioscler Thromb Vasc Biol. 1995;15(9):1512–31. 10.1161/01.atv.15.9.1512 . 7670967

[11] Schwenke DC, Carew TE. Initiation of atherosclerotic lesions in cholesterol-fed rabbits. II. Selective retention of LDL vs. selective increases in LDL permeability in susceptible sites of arteries. Arteriosclerosis. 1989;9(6):908–18. 10.1161/01.atv.9.6.908 . 2590068

[12] Berneis KK. Metabolic origins and clinical significance of LDL heterogeneity. The Journal of Lipid Research. 2002;43(9):1363–79. 10.1194/jlr.R200004-JLR200 12235168

[13] Garcia-Sanchez C, Torres-Tamayo M, Juarez-Meavepena M, Lopez-Osorio C, Toledo-Ibelles P, Monter-Garrido M, et al Lipid plasma concentrations of HDL subclasses determined by enzymatic staining on polyacrylamide electrophoresis gels in children with metabolic syndrome. Clinica chimica acta; international journal of clinical chemistry. 2011;412(3–4):292–8. 10.1016/j.cca.2010.10.021 . 21036160

[14] Goldstein JL, Ho YK, Basu SK, Brown MS. Binding site on macrophages that mediates uptake and degradation of acetylated low density lipoprotein, producing massive cholesterol deposition. Proceedings of the National Academy of Sciences of the United States of America. 1979;76(1):333–7. 10.1073/pnas.76.1.333 218198 PMC382933

[15] Rizzo M, Berneis K. Low-density lipoprotein size and cardiovascular risk assessment. QJM: monthly journal of the Association of Physicians. 2006;99(1):1–14. Epub 2005/12/24. 10.1093/qjmed/hci154 . 16371404

[16] Bolanos-Garcia VM, Soriano-Garcia M, Mas-Oliva J. Stability of the C-terminal peptide of CETP mediated through an (i, i + 4) array. Biochimica et biophysica acta. 1998;1384(1):7–15. 10.1016/s0167-4838(97)00156-8 . 9602025

[17] Mendoza-Espinosa P, Moreno A, Castillo R, Mas-Oliva J. Lipid dependant disorder-to-order conformational transitions in apolipoprotein CI derived peptides. Biochemical and biophysical research communications. 2008;365(1):8–15. Epub 2007/10/31. 10.1016/j.bbrc.2007.10.112 . 17967413

[18] Pfanzagl B. LDL oxidized with iron in the presence of homocysteine/cystine at acidic pH has low cytotoxicity despite high lipid peroxidation. Atherosclerosis. 2006;187(2):292–300. Epub 2005/11/01. 10.1016/j.atherosclerosis.2005.09.024 . 16256999

[19] Kuhn H, Heydeck D, Hugou I, Gniwotta C. In vivo action of 15-lipoxygenase in early stages of human atherogenesis. Journal of Clinical Investigation. 1997;99(5):888–93. 10.1172/JCI119253 9062346 PMC507896

[20] Manzano-Leon N, Mas-Oliva J, Sevilla-Tapia L, Morales-Barcenas R, Serrano J, MS ON, et al Particulate matter promotes in vitro receptor-recognizable low-density lipoprotein oxidation and dysfunction of lipid receptors. J Biochem Mol Toxicol. 2013;27(1):69–76. 10.1002/jbt.21452 23297186 PMC4345123

[21] Steinberg D. The LDL modification hypothesis of atherogenesis: an update. Journal of lipid research. 2009;50 Suppl:S376–81. Epub 2008/11/18. 10.1194/jlr.R800087-JLR200 19011257 PMC2674707

[22] Hansen-Hagge TE, Baumeister E, Bauer T, Schmiedeke D, Renne T, Wanner C, et al Transmission of oxLDL-derived lipid peroxide radicals into membranes of vascular cells is the main inducer of oxLDL-mediated oxidative stress. Atherosclerosis. 2008;197(2):602–11. Epub 2007/10/24. 10.1016/j.atherosclerosis.2007.08.029 . 17950298

[23] Brand K, Page S, Rogler G, Bartsch A, Brandl R, Knuechel R, et al Activated transcription factor nuclear factor-kappa B is present in the atherosclerotic lesion. Journal of Clinical Investigation. 1996;97(7):1715–22. 10.1172/JCI118598 8601637 PMC507236

[24] Navab M, Imes SS, Hama SY, Hough GP, Ross LA, Bork RW, et al Monocyte transmigration induced by modification of low density lipoprotein in cocultures of human aortic wall cells is due to induction of monocyte chemotactic protein 1 synthesis and is abolished by high density lipoprotein. Journal of Clinical Investigation. 1991;88(6):2039–46. 10.1172/JCI115532 1752961 PMC295797

[25] Vora DK, Fang ZT, Liva SM, Tyner TR, Parhami F, Watson AD, et al Induction of P-selectin by oxidized lipoproteins. Separate effects on synthesis and surface expression. Circ Res. 1997;80(6):810–8. 10.1161/01.res.80.6.810 . 9168783

[26] Stephens NG, Parsons A, Schofield PM, Kelly F, Cheeseman K, Mitchinson MJ. Randomised controlled trial of vitamin E in patients with coronary disease: Cambridge Heart Antioxidant Study (CHAOS). Lancet. 1996;347(9004):781–6. 10.1016/s0140-6736(96)90866-1 . 8622332

[27] Sundell CL, Somers PK, Meng CQ, Hoong LK, Suen KL, Hill RR, et al AGI-1067: a multifunctional phenolic antioxidant, lipid modulator, anti-inflammatory and antiatherosclerotic agent. The Journal of pharmacology and experimental therapeutics. 2003;305(3):1116–23. Epub 2003/03/11. 10.1124/jpet.102.048132 . 12626663

[28] Majesky MW, Dong XR, Hoglund V, Mahoney WM, Jr., Daum G. The adventitia: a dynamic interface containing resident progenitor cells. Arterioscler Thromb Vasc Biol. 2011;31(7):1530–9. Epub 2011/06/17. 10.1161/ATVBAHA.110.221549 21677296 PMC3382115

[29] Satoh K, Nigro P, Berk BC. Oxidative stress and vascular smooth muscle cell growth: a mechanistic linkage by cyclophilin A. Antioxid Redox Signal. 2010;12(5):675–82. 10.1089/ars.2009.2875 19747062 PMC2861539

[30] Tchivilev I, Madamanchi NR, Vendrov AE, Niu XL, Runge MS. Identification of a protective role for protein phosphatase 1cgamma1 against oxidative stress-induced vascular smooth muscle cell apoptosis. The Journal of biological chemistry. 2008;283(32):22193–205. Epub 2008/06/10. 10.1074/jbc.M803452200 18540044 PMC2494915

[31] Charles RL, Eaton P. Redox signalling in cardiovascular disease. Proteomics Clinical applications. 2008;2(6):823–36. 10.1002/prca.200780104 . 21136882

[32] Pezuk JA, Brassesco MS, Morales AG, de Oliveira JC, de Oliveira HF, Scrideli CA, et al Inhibition of polo-like kinase 1 induces cell cycle arrest and sensitizes glioblastoma cells to ionizing radiation. Cancer biotherapy & radiopharmaceuticals. 2013;28(7):516–22. Epub 2013/05/30. 10.1089/cbr.2012.1415 23713868 PMC3741430

[33] Blackwood EM, Luscher B, Kretzner L, Eisenman RN. The Myc:Max protein complex and cell growth regulation. Cold Spring Harb Symp Quant Biol. 1991;56:109–17. 10.1101/SQB.1991.056.01.015 . 1819481

[34] Portal MM, Ferrero GO, Caputto BL. N-Terminal c-Fos tyrosine phosphorylation regulates c-Fos/ER association and c-Fos-dependent phospholipid synthesis activation. Oncogene. 2007;26(24):3551–8. 10.1038/sj.onc.1210137 . 17160021

[35] Xu J, Testa JR. DLX5 (distal-less homeobox 5) promotes tumor cell proliferation by transcriptionally regulating MYC. The Journal of biological chemistry. 2009;284(31):20593–601. 10.1074/jbc.M109.021477 19497851 PMC2742824

[36] Gartel AL, Shchors K. Mechanisms of c-myc-mediated transcriptional repression of growth arrest genes. Exp Cell Res. 2003;283(1):17–21. 10.1016/s0014-4827(02)00020-4 . 12565816

[37] Kular L, Pakradouni J, Kitabgi P, Laurent M, Martinerie C. The CCN family: a new class of inflammation modulators? Biochimie. 2011;93(3):377–88. 10.1016/j.biochi.2010.11.010 . 21130134

[38] Birkedal-Hansen H, Moore WG, Bodden MK, Windsor LJ, Birkedal-Hansen B, DeCarlo A, et al Matrix metalloproteinases: a review. Crit Rev Oral Biol Med. 1993;4(2):197–250. Epub 1993/01/01. . 8435466 10.1177/10454411930040020401

[39] Gerthoffer WT. Mechanisms of vascular smooth muscle cell migration. Circ Res. 2007;100(5):607–21. 10.1161/01.RES.0000258492.96097.47 . 17363707

[40] Kaminski WE, Wenzel JJ, Piehler A, Langmann T, Schmitz G. ABCA6, a novel a subclass ABC transporter. Biochemical and biophysical research communications. 2001;285(5):1295–301. 10.1006/bbrc.2001.5326 . 11478798

[41] Piehler A, Kaminski WE, Wenzel JJ, Langmann T, Schmitz G. Molecular structure of a novel cholesterol-responsive A subclass ABC transporter, ABCA9. Biochemical and biophysical research communications. 2002;295(2):408–16. 10.1016/s0006-291x(02)00659-9 . 12150964

[42] Levitan I, Volkov S, Subbaiah PV. Oxidized LDL: diversity, patterns of recognition, and pathophysiology. Antioxid Redox Signal. 2010;13(1):39–75. 10.1089/ars.2009.2733 19888833 PMC2877120

[43] Jimenez-Corona AE, Damian-Zamacona S, Perez-Torres A, Moreno A, Mas-Oliva J. Osteopontin upregulation in atherogenesis is associated with cellular oxidative stress triggered by the activation of scavenger receptors. Archives of medical research. 2012;43(2):102–11. Epub 2012/03/14. 10.1016/j.arcmed.2012.03.001 . 22410136

[44] Zheng JY, Koda T, Fujiwara T, Kishi M, Ikehara Y, Kakinuma M. A novel Rab GTPase, Rab33B, is ubiquitously expressed and localized to the medial Golgi cisternae. J Cell Sci. 1998;111 (Pt 8):1061–9. . 9512502 10.1242/jcs.111.8.1061

[45] Itoh T, Fujita N, Kanno E, Yamamoto A, Yoshimori T, Fukuda M. Golgi-resident small GTPase Rab33B interacts with Atg16L and modulates autophagosome formation. Molecular biology of the cell. 2008;19(7):2916–25. Epub 2008/05/02. 10.1091/mbc.E07-12-1231 18448665 PMC2441679

[46] Miller AM, Asquith DL, Hueber AJ, Anderson LA, Holmes WM, McKenzie AN, et al Interleukin-33 induces protective effects in adipose tissue inflammation during obesity in mice. Circ Res. 2010;107(5):650–8. 10.1161/CIRCRESAHA.110.218867 20634488 PMC4254700

[47] Weber C, Noels H. Atherosclerosis: current pathogenesis and therapeutic options. Nature medicine. 2011;17(11):1410–22. Epub 2011/11/09. 10.1038/nm.2538 . 22064431

[48] Guo J, Li L, Wu YJ, Yan Y, Xu XN, Wang SB, et al Inhibitory effects of Brazilin on the vascular smooth muscle cell proliferation and migration induced by PDGF-BB. Am J Chin Med. 2013;41(6):1283–96. 10.1142/S0192415X13500869 . 24228601

[49] Bochkov VN, Leitinger N. Anti-inflammatory properties of lipid oxidation products. Journal of molecular medicine (Berlin, Germany). 2003;81(10):613–26. 10.1007/s00109-003-0467-2 . 13679995

[50] Rama P, Matuska S, Paganoni G, Spinelli A, De Luca M, Pellegrini G. Limbal stem-cell therapy and long-term corneal regeneration. The New England journal of medicine. 2010;363(2):147–55. 10.1056/NEJMoa0905955 . 20573916

[51] Minta J, Jungwon Yun J, St Bernard R. Microarray analysis of ox-LDL (oxidized low-density lipoprotein)-regulated genes in human coronary artery smooth muscle cells. Cell Biol Int Rep (2010). 2010;17(2):e00007 10.1042/CBR20100006 23119143 PMC3475437

[52] Christen T, Bochaton-Piallat ML, Neuville P, Rensen S, Redard M, van Eys G, et al Cultured porcine coronary artery smooth muscle cells. A new model with advanced differentiation. Circ Res. 1999;85(1):99–107. 10.1161/01.res.85.1.99 . 10400915

[53] Patel S, Shi Y, Niculescu R, Chung EH, Martin JL, Zalewski A. Characteristics of coronary smooth muscle cells and adventitial fibroblasts. Circulation. 2000;101(5):524–32. 10.1161/01.cir.101.5.524 . 10662750

[54] Chistiakov DA, Orekhov AN, Bobryshev YV. Vascular smooth muscle cell in atherosclerosis. Acta Physiol (Oxf). 2015;214(1):33–50. 10.1111/apha.12466 . 25677529

[55] Rensen SS, Doevendans PA, van Eys GJ. Regulation and characteristics of vascular smooth muscle cell phenotypic diversity. Neth Heart J. 2007;15(3):100–8. 10.1007/bf03085963 17612668 PMC1847757

[56] Chatterjee S. Role of oxidized human plasma low density lipoproteins in atherosclerosis: effects on smooth muscle cell proliferation. Molecular and cellular biochemistry. 1992;111(1–2):143–7. 10.1007/bf00229586 . 1588938

[57] Felty Q, Xiong WC, Sun D, Sarkar S, Singh KP, Parkash J, et al Estrogen-induced mitochondrial reactive oxygen species as signal-transducing messengers. Biochemistry. 2005;44(18):6900–9. 10.1021/bi047629p . 15865435

[58] Morrissey JJ, Klahr S. Agmatine activation of nitric oxide synthase in endothelial cells. Proc Assoc Am Physicians. 1997;109(1):51–7. . 9010916

[59] Mesquita FS, Dyer SN, Heinrich DA, Bulun SE, Marsh EE, Nowak RA. Reactive oxygen species mediate mitogenic growth factor signaling pathways in human leiomyoma smooth muscle cells. Biol Reprod. 2010;82(2):341–51. 10.1095/biolreprod.108.075887 19741209 PMC2809225

[60] Tsimikas S. Oxidative biomarkers in the diagnosis and prognosis of cardiovascular disease. Am J Cardiol. 2006;98(11A):9P–17P. 10.1016/j.amjcard.2006.09.015 . 17126679

[61] Galkina E, Ley K. Immune and inflammatory mechanisms of atherosclerosis (*). Annu Rev Immunol. 2009;27:165–97. 10.1146/annurev.immunol.021908.132620 19302038 PMC2734407

[62] Ross R. Atherosclerosis is an inflammatory disease. Am Heart J. 1999;138(5 Pt 2):S419–20. 10.1016/S0002-8703(99)70266-8 . 10539839

[63] Aguilar-Gaytan R, Mas-Oliva J. Oxidative stress impairs endocytosis of the scavenger receptor class A. Biochemical and biophysical research communications. 2003;305(3):510–7. 10.1016/S0006-291x(03)00796-4 . 12763022

[64] Innerarity TL, Pitas RE, Mahley RW. Lipoprotein-receptor interactions. Methods Enzymol. 1986;129:542–65. 10.1016/0076-6879(86)29091-6 . 2425225

[65] Mahley RW, Innerarity TL, Weisgraber KH, Fry DL. Canine hyperlipoproteinemia and atherosclerosis. Accumulation of lipid by aortic medial cells in vivo and in vitro. Am J Pathol. 1977;87(1):205–26. 192082 PMC2032077

[66] Pitas RE, Innerarity TL, Mahley RW. Cell surface receptor binding of phospholipid. protein complexes containing different ratios of receptor-active and -inactive E apoprotein. The Journal of biological chemistry. 1980;255(11):5454–60. . 7372644

[67] Pitas RE, Innerarity TL, Weinstein JN, Mahley RW. Acetoacetylated lipoproteins used to distinguish fibroblasts from macrophages in vitro by fluorescence microscopy. Arteriosclerosis. 1981;1(3):177–85. 10.1161/01.atv.1.3.177 . 6895305

